# A State Home for Inebriates

**Published:** 1902-04

**Authors:** 


### A STATE HOME FOR INEBRIATES.

DR. WILLIS B. PARKS, of this city, in a communication to the *Atlanta Journal*, urges the establishment of an institution for the cure of inebriety, to be maintained by the State. The communication received highly commendatory editorial comment. The time has passed, or is rapidly passing, when the unfortunate alcoholic is regarded as a semi-criminal, and when the blue-nose bum at the corner saloon was looked at with no other emotion than that of aversion and disgust. Advancing hand in hand with science, humanitarianism has shown us that these unfortunates are often the bounden slaves of transmitted habit, and that they are unable, without the interposition of a rescuing hand, to cope with a perversion which dominates an already weakened and inefficient will-power. Such are worthy objects for State aid. Instead of regarding them with averted head as inevitable consequences of the vice of civilization, victims of voluntary assumption of an enslaving habit which began as pleasure and ended in grimmest tragedy, it is manifest that these wretched beings are brought to this condition in obedience to a law that is more potent than their resistance, and that they must be sheltered and protected in the same manner as are the insane and mental defectives. It is equally manifest that this purpose cannot be attained by prayer or appeal to the intellectual nature. It is only now and then that an unfortunate is reclaimed, or rescued, as the term is, and braces up and voluntarily relinquishes his habit of intemperance. The moral nature is too apt to be atrophied, while the intellectual is a very fluctuating quantity. Reformation from evil habits requires bracing at every advance, else there will be the inevitable backsliding and a return to a condition that is worse than the first. Institutional treatment for alcoholism is the most rational and the most successful. Removal of the habitue from his environment, securing the atmosphere conducive to success, with proper control and the administration of the appropriate drug remedies, are advantages that are offered by insiitutions.

The treatment of the drink habit is too large a subject to discuss in this place, further than to note that home treatment is markedly inferior in results to those achievedin institutions.

There seems to be a tendency for secret vices to seek secret cures, and this phase of human nature is responsible for the exploitation of divers systems and nostrums of greater or less renown, and of which the proprietors are influenced by no higher motive than extracting money to the limit. This does not apply to many honest physicians who are doing excellent work in the cure of inebriety, but they are numerically vastly outstripped by the quacks. A State institution would hamper the freedom of quacks and quack establishments, and exercise a paternal and beneficent influence over the weak and unwary without the idea of imprisonment or bearing the stigma of an insane asylum. The sole objection, if it may be so termed, to the foundation of a State institution for inebriates, is that the majority of drinkers are loth to confess to the drink habit, and would, therefore, be unlikely to take advantage of the benefits of the institution unless a certain amount of compulsion were exercised. It would then be left to the open and unabashed drunkards to reap whatever of good remained in life in the way of possible restoration to health and usefulness.

